# Using CRISPRa and CRISPRi Technologies to Study the Biological Functions of ITGB5, TIMP1, and TMEM176B in Prostate Cancer Cells

**DOI:** 10.3389/fmolb.2021.676021

**Published:** 2021-05-24

**Authors:** Yi Yang, Qingxing Feng, Kun Hu, Feng Cheng

**Affiliations:** ^1^ Department of Urology, Shenzhen Second People’s Hospital, the First Affiliated Hospital of Shenzhen University, Shenzhen, China; ^2^ Deparment of Urology, Institute of Surgery Research, Daping Hospital, Third Military Medical University, Chongqing, China; ^3^ Anhui Medical University, Hefei, China

**Keywords:** prostate cancer, ITGB5, TIMP1, TMEM176B, CRISPR interference, CRISPR activation

## Abstract

Although ITGB5, TIMP1, and TMEM176B are abnormally expressed in several cancers, their molecular biological mechanisms in prostate cancer cells are still to be investigated. The gene regulation technogies based on CRISPR transcription factors could be used to investigate the biological functions of genes in cancer. In this study, we used CRISPR interference (CRISPRi) and CRISPR activation (CRISPRa) technologies to regulate the transcription of ITGB5, TIMP1, and TMEM176B in prostate cancer cells. Through a series of cellualr experiments, we found that inhibition of ITGB5 or activation of TIMP1 and TMEM176B suppress prostate cancer. The three genes synergistically affect the proliferation, invasion and migration capabilities of cancer cells.

## Introduction

Prostate cancer is one of the most common malignant tumors in the genitourinary system. The incidence rate of prostate cancer ranks the second among all kinds of malignant tumors worldwide (Neupane et al., 2017). The incidence rate of prostate cancer is the highest in Europe and the United States, and its mortality rate is only less than lung cancer (Siegel et al., 2018). In China, with the acceleration of population aging, changes in diet structure and improvement of medical level, the incidence rate of prostate cancer has also increased rapidly (Zhang et al., 2009). Prostate cancer tends to occur in elderly men, but in recent years there is a trend of younger. For early diagnosis of prostate cancer, radical surgery or radiotherapy can achieve curative effect, and its 5-years survival rate is almost 100%. However, for advanced metastatic prostate cancer, the 5-years survival rate was only 34% (Preston, 2009). Therefore, early detection and diagnosis of prostate cancer is particularly important to improve the survival rate of patients after treatment.

The study on early diagnostic markers of prostate cancer has been a hot topic in recent years. At the same time, the clinical efficacy of advanced prostate cancer, especially castration resistant prostate cancer, is still very limited. New therapeutic methods still depend on the development and utilization of drug target molecules. The molecular biological mechanism of prostate cancer is also a hot issue in the research of urinary tumor.

ITGB5, TIMP1, and TMEM176B are abnormally expressed in several human cancers. Among them, ITGB5 is highly expressed cancers, TIMP1, and TMEM176B are lowly expressed in cancers. ITGB5 (Integrin β5, Integrin β5) is one of the integrin family members. As a type of transmembrane glycoprotein located on the cell surface, integrins are mainly dimers composed of *α* and *β* subunits (Alavi et al., 2003; Hood et al., 2003). Recently, there is new evidence that ITGB5 can promote cell migration and invasion Bianchi et al. (2010), and may participate in the epithelial-mesenchymal transition. The epithelial-mesenchymal transition plays an extremely important role in tumor metastasis. It is found that ITGB5 promotes the lymph node metastasis of colorectal cancer (Denadai et al., 2013). Bianchi et al. found that over-expression of ITGB5 promotes the differentiation and mutation of breast cancer cells and makes breast cancer more aggressive (Bianchi et al., 2010). The protein encoded by TIMP1 is a natural inhibitor of matrix metalloproteinases which are a group of peptidases involved in the degradation of extracellular matrix. TIMP1 is thus closely related to tumor invasion and metastasis. TMEM176B is a transmembrane protein that regulates the immune response of the tumor microenvironment. Although these evidences suggest that ITGB5, TIMP1, and TMEM176B may work together to promote the invasion and metastasis of cancer cells, their molecular biological mechanisms in prostate cancer cells are still unclear and require further research.

In this work, we have used CRISPR interference (CRISPRi) and CRISPR activation (CRISPRa) technologies to study the biologocal functions of ITGB5, TIMP1, and TMEM176B in prostate cancer cells. We found that ITGB5 promotes cancer, while TIMP1 and TMEM176B suppress cancer. The three genes synergistically affect the proliferation, invasion, and migration capabilities of prostate cancer cells.

## Materials and Methods

### Cell Culture

LNCap cells were obtained from The Cell Bank of Chinese Academy of Sciences (Shanghai, China). The cells were cultured in DMEM (HyClone, United States) added with 100 U/ml penicillin/streptomycin and 15% fetal bovine serum (FBS; Gibco, United States) under a moisturized atmosphere with a 5% CO_2_ level.

### CRISPR Interference and CRISPR Activation System

The CRISPR-dCas9-VPR and CRISPR-dCas9-KRAB plasmids were ordered from Addgene (Cambridge, MA, United States). The sgRNAs targeting ITGB5, TIMP1, and TMEM176B were designed by the online software CHOPCHOP, a CRISPR/Cas9, and TALEN web tool for genome editing.

### Cell Transfection

LNCap cells were seeded in 6-well plates with a 3 × 10^6^ cells per well density in the environment of DMEM. Followed the manufacturer’s protocol of Lipofectamine 2000 Transfection Kit (Thermo Fisher Scientific, Waltham, MA, United States), transfection was conducted. After that, the cells were set for 48 h in order for future analysis.

### Quantitative Real-Time Polymerase Chain Reaction

At 48 h after gene transfection, TRIzol (Invitrogen) was used as a separating reagent for total RNA to get the first-strand cDNA using a FastKing RT Kit (With gDNase) (Tiangen) following the official instructions. The qRT-PCR analysis was carried out by SYBR Green (Tiangen). GAPDH was taken as endogenous controllers for the three mRNAs.

### Cell Proliferation Assay

LNCap cells were seeded in a 96-well plate at a 1 × 10^5^/ml dose and incubated for 24 h. Subsequently, cells were starved for another 2 h. Culture medium was replaced with 20 μL of methyl thiazolyl tetrazolium (MTT) solution (5 g/L) (Sigma-Aldrich, St. Louis, MO, United States) and incubated at 37°C for 4 h. The supernatant was discarded and 100 μL of DMSO (dimethyl sulfoxide) (Sigma-Aldrich, St. Louis, MO, United States) was put on to each well. The absorbance value was tracked at the wavelength of 570 nm with a microplate reader.

### Cell Apoptosis Assay

At 48 h after cell transfection, the apoptosis of LNCap cells was determined by the Caspase-3 Assay Kit (Fluorometric) (ab39383). Caspase-3 activity was detected by spectrophotometry and each assay was performed in triple.

### Cell Migration Assay

LNCap cells were treated with 1.8 mmol/L hydroxyurea for 12 h to inhibit cell proliferation and then a 100 μL pipette tip was used to make cell scratches in a vertical well plate. Then the cell culture medium was discarded and washed three times with PBS. Then the cells were culture and taken pictures. The 0 and 24 h pictures were recorded, and the imageproplus 6.0 software was used to analyze and calculate the cell migration distance.

### Cell Invasion Assay

After 48 h of transfection, cells from each group were taken, trypsinized, and then inoculated in a matrigel-Tran-swell chamber. The serum-free DMEM medium was used for culture in the chamber. 10% FBS culture medium was added to the lower chamber. After 24 h, the upper uninvaded cells was wipped with a cotton swab. After fixing with 4% polymethanol for 15 min, cells were stained with 1% crystal violet for 5 min. After washing with PBS for three times, five fields under the fluorescence microscope were randomly selected and observed, and the invaded cells were counted and recorded.

### Statistical Analysis

The data were expressed as means ± standard deviations (SD). The SPSS version 19.0 software (IBM Corporation, United States) was used for statistical analysis. A *p*-value < 0.05 was considered statistically significant.

## Results

### Expression of ITGB5, TIMP1, TMEM176B in Prostate Cancer Cells

**FIGURE 1:**
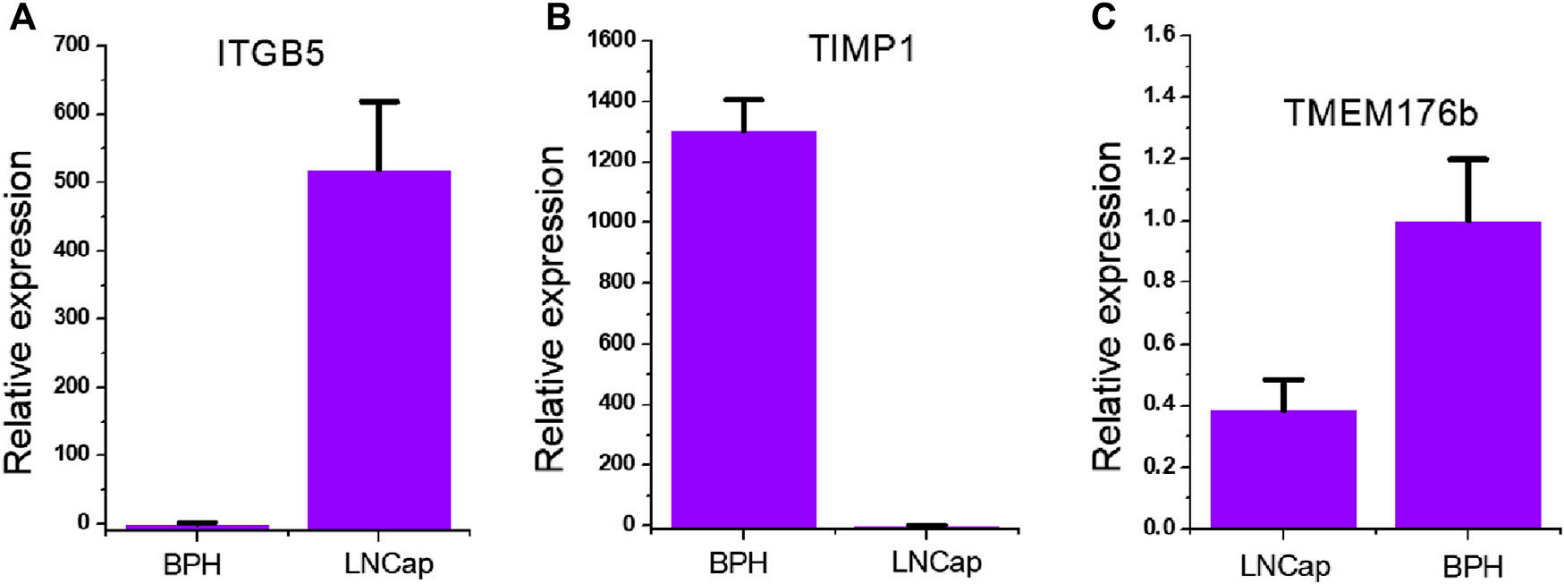
Expression of ITGB5, TIMP1, TMEM176B in prostate cancer cells. **(A)** qRT-PCR analysis of ITGB5 expression in LNCap and BPH cell lines. **(B)** qRT-PCR analysis of TIMP1expression in LNCap and BPH cell lines. **(C)** qRT-PCR analysis of TMEM176Bexpression in LNCap and BPH cell lines.

In order to clarify the overall expression patterns of the three genes in prostate cancer, we tested the expression of ITGB5 (Figure 1A), TIMP1 (Figure 1B), and TMEM176B (Figure 1C) in the prostate cancer LNCap cell line. We found that ITGB5 was significantly higher in prostate cancer compared with BPH cells, and that TMP1 and TMEM176B were significantly low expressed in prostate cancer. This suggests that ITGB5 is a potential proto-oncogene in the prostate cancer LNCap cell line, while TMP1 and TMEM176B are potential tumor suppressor genes.

### The Effects of Knockdown of ITGB5 or Activation of TIMP1 and TMEM176B Expression on Cell Proliferation and Apoptosis

**FIGURE 2:**
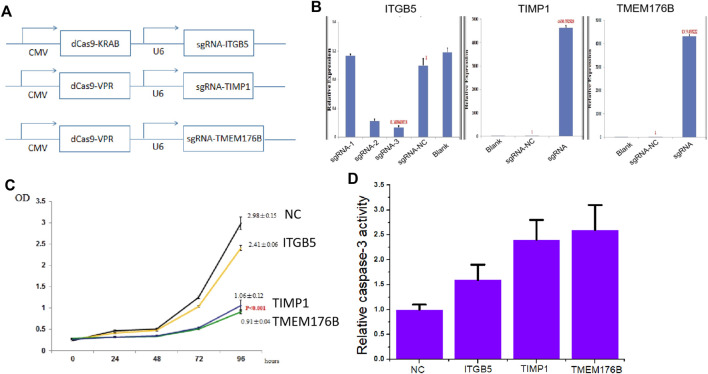
The effects of knockdown of ITGB5 or activation of TIMP1 and TMEM176B expression on cell proliferation and apoptosis. **(A)** Vector maps of CRISPR-KRAB and CRISPR-VPR plasmids constructed in this work. **(B)** LNCap cells were transfected by CRISPRi or CRISPRa systems and gene expression was confirmed by real-time PCR. **(C)** MTT assays revealed that knockdown of ITGB5 or activation of TIMP1 and TMEM176B expression inhibited the growth rate of LNCap cells. **(D)** ELISA assays revealed that knockdown of ITGB5 or activation of TIMP1 and TMEM176B expression increased the apoptosis rate of LNCap cells.

In order to further clarify whether ITGB5 is a proto-oncogene and whether TIMP1 and TMEM176B are tumor suppressor genes, we used dCas9-KRAB-mediated CRISPRi technology (Figure 2A) to knock down the expression of ITGB5 (Figure 2B), and used dCas9-VPR-mediated CRISPRa technology (Figure 2A) to activate TIMP1 and TMEM176B expression (Figure 2B). The experimental results of MTT show that inhibiting the expression of ITGB5 can reduce cell proliferation, while activating the expression of TIMP1 and TMEM176B can achieve the same effect (Figure 2C). In addition, the results of Caspase-3 ELISA assay show that inhibiting the expression of ITGB5 can increase cell apoptosis, while activating the expression of TIMP1 and TMEM176B can observe the same effect (Figure 2D).

### The Effects of Knockdown of ITGB5 or Activation of TIMP1 and TMEM176B Expression on Cell Migration

**FIGURE 3:**
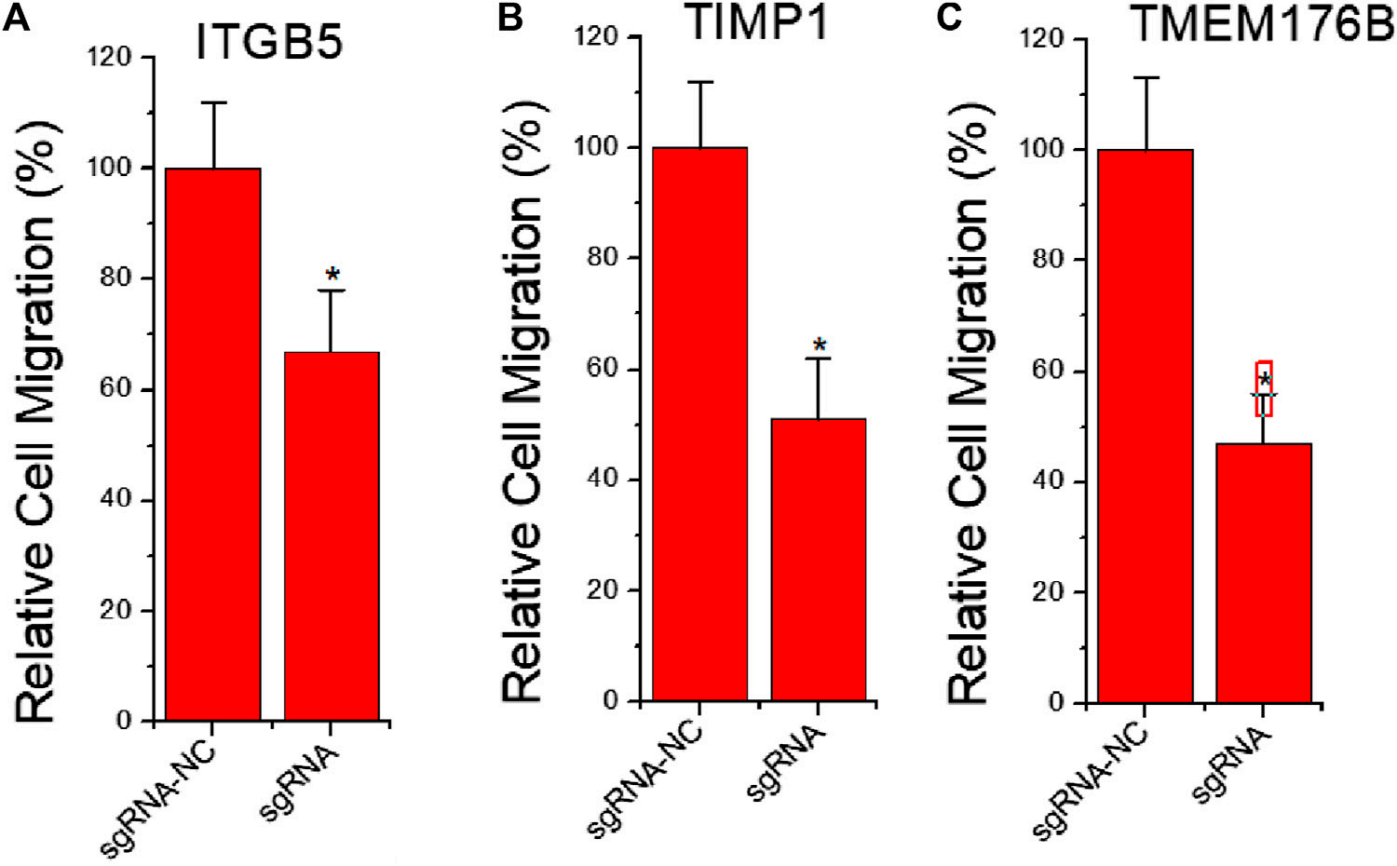
The effects of knockdown of ITGB5 or activation of TIMP1 and TMEM176B expression on cell migration. LNCap cells were transfected by CRISPRi or CRISPRa systems and cell migration assay revealed that knockdown of ITGB5 **(A)** or activation of TIMP1 **(B)** and TMEM176B **(C)** expression inhibited the migration rate of LNCap cells.

To further clarify whether inhibition of ITGB5 and activation of TIMP1 and TMEM176B affect tumor cell migration, we also used dCas9-KRAB-mediated CRISPRi technology to knock down ITGB5 expression, and used dCas9-VPR-mediated CRISPRa technology to induce TIMP1 and TMEM176B activation. The results of the cell scratch experiment showed that inhibiting the expression of ITGB5 can reduce cell migration viability, while activating the expression of TIMP1 and TMEM176B can achieve similar effects (Figures 3A,B).

### The Effects of Knockdown of ITGB5 or Activation of TIMP1 and TMEM176B Expression on Cell Invasion

**FIGURE 4:**
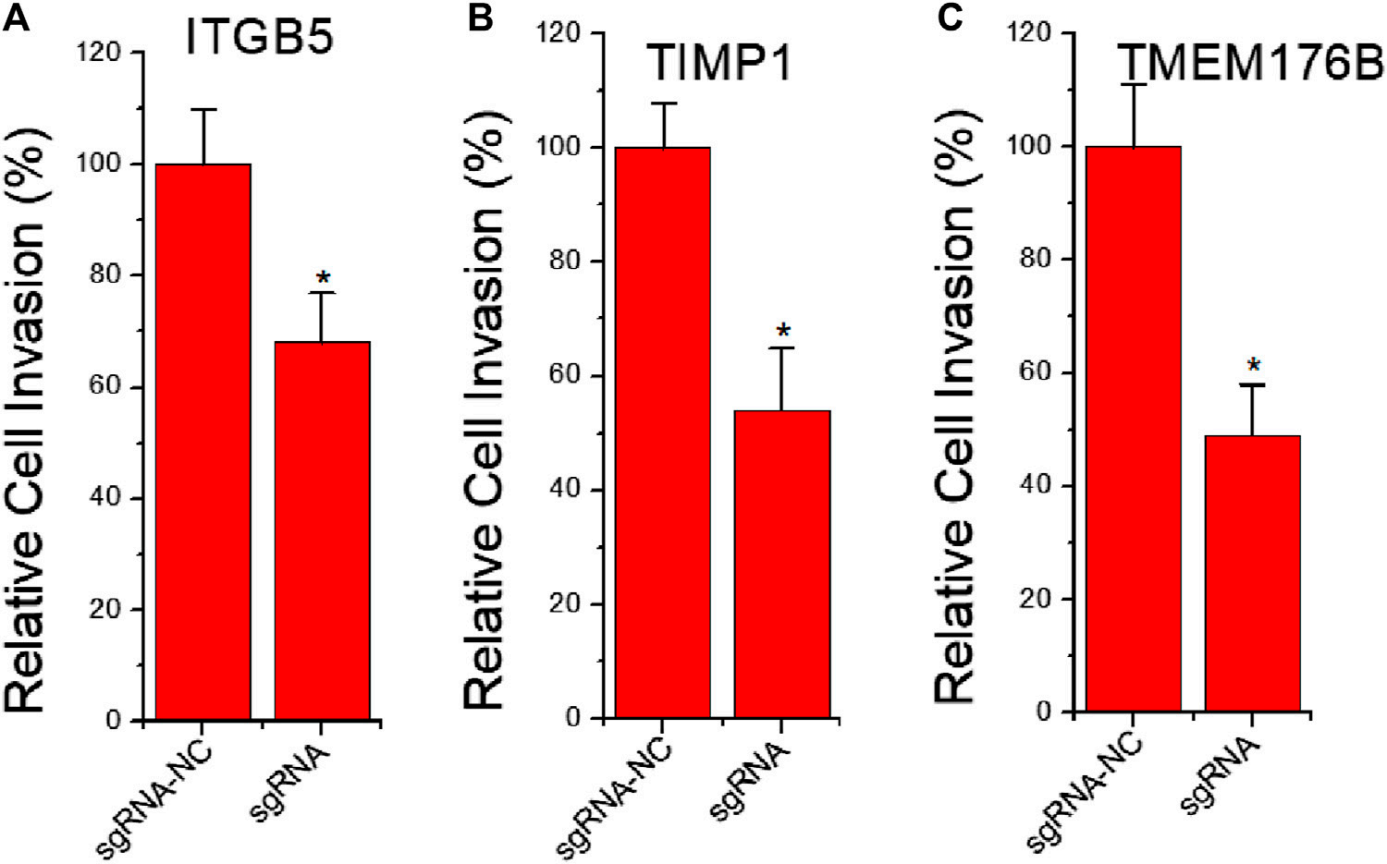
The effects of knockdown of ITGB5 or activation of TIMP1 and TMEM176B expression on cell invasion. LNCap cells were transfected by CRISPRi or CRISPRa systems and cell migration assay revealed that knockdown of ITGB5 **(A)** or activation of TIMP1 **(B)** and TMEM176B **(C)** expression inhibited the invasion rate of LNCap cells.

Cell migration is always directly related to invasion behavior. To further clarify whether knockdown of ITGB5 and activation of TIMP1 and TMEM176B expression affect tumor cell invasion, we used dCas9-KRAB-mediated CRISPRi to knock down ITGB5 expression, and used dCas9-VPR-mediated CRISPRa to activate TIMP1 and TMEM176B expression. The results of cell invasion experiments show that inhibiting the expression of ITGB5 can reduce the ability of cell invasion, while activating the expression of TIMP1 and TMEM176B can achieve similar effects (Figures 4A,B).

## Discussion

Previous studies on gene function have often focused on individual genes and lacked overall understanding of tumors. A convenience provided by the advancement of CRISPR technology is that by fusing dCas9 with different transcriptional regulators, we can easily activate and reduce the expression of different genes (Morgens et al., 2016). In this way, the research on the biological functions of genes can be quickly promoted. In the past, RNA interference technology represented by siRNA tends to have low gene knock-down efficiency and high off-target efficiency (Fakhr et al., 2016). The overexpression gene technology can only express genes with smaller coding fragments (Capriotti et al., 2020). So there is a rapid progress.

In this study, we found that compared with prostate hyperplasia cells BPH, ITGB5 is significantly higher expressed in prostate cancer cells, and TMP1 and TMEM176B are significantly lower expressed. We designed different CRISPR transcriptional regulatory systems to silence ITGB5 in prostate cancer cells and activate TIMP1 and TMEM176B at the same time. MTT experiments, cell invasion and migration experiments proved that knocking down ITGB5 or overexpressing TIMP1 and TMEM176B can significantly inhibit the proliferation, invasion and migration ability of LNCap cells. The three genes synergistically affect the proliferation, invasion and migration capabilities of cancer cells. ITGB5, TIMP1, and TMEM176B can be used as molecular diagnostic targets or therapeutic targets in the future, as their combination is useful for cancer treatment. Furthermore, we can also use AAV to deliver the CRISPR system to target and regulate these three genes for gene therapy of prostate cancer.

## Data Availability

The original contributions presented in the study are included in the article/Supplementary Material, further inquiries can be directed to the corresponding author.
